# Computed tomography sheds new light on the affinities of the enigmatic euarthropod *Jianshania furcatus* from the early Cambrian Chengjiang biota

**DOI:** 10.1186/s12862-020-01625-4

**Published:** 2020-06-01

**Authors:** Yu Liu, Javier Ortega-Hernández, Hong Chen, Huijuan Mai, Dayou Zhai, Xianguang Hou

**Affiliations:** 1 Yunnan Key Laboratory for Palaeobiology, Institute of Palaeontology, Yunnan University, Kunming, 650500 China; 2 MEC International Joint Laboratory for Palaeobiology and Palaeoenvironment, Yunnan University, Kunming, 650500 China; 3 Museum of Comparative Zoology and Department of Organismic and Evolutionary Biology, Harvard University, 26 Oxford Street, Cambridge, MA 02138 USA

**Keywords:** Euarthropoda, Konservat-Lagerstätte, Exceptional preservation, Pyritization, Fuxianhuiid, Computed tomography

## Abstract

**Background:**

The Chengjiang biota is one of the most species-rich Cambrian Konservat-Lagerstätten, and preserves a community dominated by non-biomineralized euarthropods. However, several Chengjiang euarthropods have an unfamiliar morphology, are extremely rare, or incompletely preserved.

**Results:**

We employed micro-computed tomography to restudy the enigmatic euarthropod *Jianshania furcatus*. We reveal new morphological details, and demonstrate that the specimens assigned to this species represent two different taxa. The holotype of *J. furcatus* features a head shield with paired anterolateral notches, stalked lateral eyes, and an articulated tailspine with a bifurcate termination. The other specimen is formally redescribed as *Xiaocaris luoi* gen. et sp. nov., and is characterized by stalked eyes connected to an anterior sclerite, a subtrapezoidal head shield covering three small segments with reduced tergites, a trunk with 15 overlapping tergites with a well-developed dorsal keel, and paired tail flukes.

**Conclusions:**

The presence of antennae, biramous appendages with endopods composed of 15 articles, and multiple appendage pairs associated with the trunk tergites identify *X. luoi* nov. as a representative of Fuxianhuiida, an early branching group of stem-group euarthropods endemic to the early Cambrian of Southwest China. *X. luoi* nov. represents the fifth fuxianhuiid species described from the Chengjiang biota, and its functional morphology illuminates the ecological diversity of this important clade for understanding the early evolutionary history of euarthropods.

## Background

**Fig. 1:**
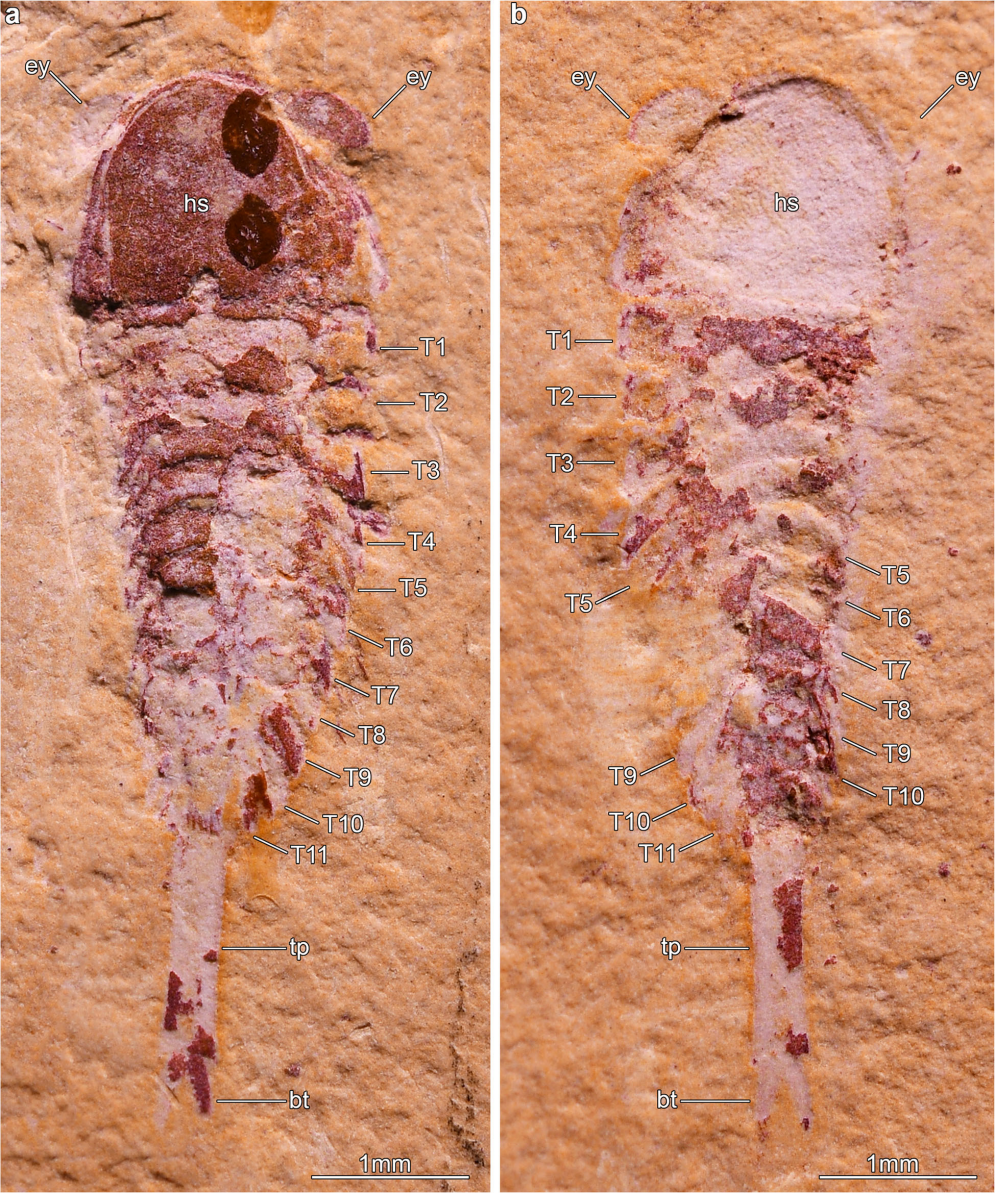
*Jianshania furcatus*, from the Ercaicun section of Haikou, Cambrian (Stage 3) Chengjiang biota. **a** Part of holotype (Hz-f-6-307a), complete specimen preserved in dorsal–ventral aspect, photographed under cross-polarized light. See also [41], Plate 7, Fig. 4. **b** Counterpart of holotype (Hz-f-6-307b), photographed under cross-polarized light

The Yangtze Platform in China contains several Cambrian localities that have produced critical insights into the origin and early evolution of complex organisms, as well as the ecological dynamics of some of the earliest animal-dominated ecosystems [1, 2]. In addition to a substantial shelly fossil record [3, 4], this large area is world-renowned due to the occurrence of numerous sites containing soft-tissue fossil preservation, or Konservat-Lagerstätten, that have fundamentally illuminated the extinct biodiversity at the time in a variety of marine settings during the Cambrian Explosion. Exceptional deposits in the Yangtze Platform are geographically and stratigraphically widespread for the Cambrian, with sites ranging from Stage 3 (e.g. Chengjiang, Qingjiang, Xiaoshiba; see [2, 3, 5, 6], to Stage 4 (e.g. Balang, Guanshan, Shipai; see [7–9]), Wuliuan (Kaili; see [10]) and even Furongian (Guole; see [11]). However, the Chengjiang biota of the Yu’anshan Member, Chiungchussu Formation, of Yunnan Province distinguishes itself from other fossiliferous localities in China by its unparalleled species richness, quality of fossil preservation, abundance and geographical coverage [3, 12], to the extent that it represents the only confirmed Tier 1 Burgess Shale-type deposit in the Yangtze Platform to date [13]. Since its discovery in 1984, the Chengjiang biota has been a focal point of intense palaeobiological scrutiny due to the abundance of non-biomineralized macrofossils resulting from organic carbon preservation and decay-induced pyritization by sulphur-reducing bacteria [14, 15]. Similar to other major Cambrian Konservat-Lagerstätten around the world [16–20], the metazoan community preserved in Chengjiang is dominated by euarthropods in terms of species richness and abundance, which in addition to a rich trilobite fauna also includes several dozens of non-biomineralized taxa [12, 21, 22]. The superb quality of soft-tissue preservation in Chengjiang fossils has produced deep insights into the biology of Cambrian euarthropods, informing about important aspects of their internal anatomy such as the digestive tract [23–25] and nervous system [26–28], as well as insights into their behavior and ecology [29–31]. Arguably one of the most remarkable contributions of Chengjiang euarthropods has been the availability of detailed morphological data on the ventral appendages of disparate groups that illuminate the functional morphology and higher phylogenetic affinities of these organisms [29, 32–34]. More recently, the application of X-ray based computed tomography has – somewhat metaphorically – opened a new dimension to the study of Chengjiang euarthropods [35]. Computed tomography allows to recover minute and detailed information on the three-dimensional organization of the exoskeleton and appendages that would normally be concealed within the rock matrix, without the need for mechanical preparation that would risk damaging the specimens [36–40]. One of the main advantages of this approach is that it allows restudying poorly known taxa that are extremely rare, and hence precious within the Chengjiang biota. Such is the case of *Jianshania furcatus* [41], Fig. 1 described from the Ercaicun section in Haikou, Kunming. Originally regarded as a problematic euarthropod of uncertain affinity, the overall exoskeletal anatomy, appendicular organization and significance of *Jianshania* have remained completely overlooked for more than 20 years, with the exception of cursory mentions in a few studies exploring taxonomic diversity of soft-bodied fossils in Cambrian deposits in South China [3, 12, 16, 22, 42]. Here, we employ micro-CT to reveal previously unknown details of the dorsal exoskeleton and ventral appendage organization of *Jianshania furcatus*, and demonstrate that the type material actually encompasses two distinct species that contribute towards a better understanding of the diversity of Cambrian euarthropods in the Chengjiang biota.

## Results

### Systematic Palaeontology

Euarthropoda Lankester, 1904 [43].

Deuteropoda Ortega-Hernández, 2016 [44].

Fuxianhuiida Bousfield, 1995 [45].

### Constituent taxa

*Liangwanghania biloba* [46]; *Shankouia zhenghei* [47]; Fuxianhuiidae [21] (including *Fuxianhuia protensa* [48]; *Fuxianhuia xiaoshibaensis* [5]; *Guangweicaris spinatus* [49]; *Xiaocaris luoi* nov.); Chengjiangocarididae [21] (including *Chengjiangocaris longiformis* [49, 50]; *Chengjiangocaris kunmingensis* [5]; *Alacaris mirabilis* [51]). Modified from [51].

### Diagnosis

Euarthropods with a subtrapezoidal head shield articulated with an anterior sclerite carrying stalked compound eyes. Trunk with broadly overlapping tergites that taper in width posteriorly. Variable number of anteriormost reduced tergites, completely or partially concealed under head shield. Pre-oral first appendage pair antenniform, composed of up to 20 articles. Para-oral second appendage robust and uniramous, consisting of three articles with acute termination. Sclerotized hypostome covering mouth opening and proximal bases of second appendage pair. Where known, hypostome with lateral wing-like extensions, and median notched posterior margin. Post-oral trunk appendages biramous, with a homonomous construction. Each of the anteriormost reduced tergites corresponds to one pair of appendages, while each normal trunk tergite corresponds to up to four biramous appendage pairs. Endopod consisting of at least 12 articles. Differentiated gnathobasic protopodite confirmed in some species. Exopod oval-shaped, fringed with short marginal setae. Tailspine conical or paddle-shaped, associated with paired tail flukes. Modified from [51].

### Remarks

Fuxianhuiids have some unusual traits that can cause confusion when describing their preserved morphology. The organization of the metameric units that constitute the body offers clear evidence of dorsoventral segmental mismatch between the exoskeleton, appendages, and even the internal organs [5, 21, 25, 31, 51–55]. The fossilized fuxianhuiid body consists of a variable number of broadly articulating tergites (i.e. dorsal exoskeletal plates), and these can be associated with between one and four pairs of ventral biramous appendages depending on their position within the trunk, as well as the taxon itself. For instance, whereas the anterior small segments with reduced tergites found underneath the head shield carry only one pair of appendages in all fuxianhuiids, the full-sized tergites that constitute most of the trunk are associated with two appendage pairs in *Fuxianhuia* [21, 31] and *Guangweicaris* [52], and up to four appendage pairs in *Chengjiangocaris* [5] and *Alacaris* [51], implying the presence of multiple segmental units per tergite. External segmental mismatch has been observed between the number of dorsal tergites and ventral appendage pairs; however, the segmental organization of sternites (i.e. ventral exoskeletal plates) is uncertain, as these structures are not known from any fuxianhuiid described to date. Because of this derived condition, we describe the preserved exoskeletal trunk organization of fuxianhuiids based on the number and morphology of the tergites for clarity, and only make reference to the underlying segments in cases where there is no evidence for dorsoventral mismatch, such as the anterior small segments underneath the head shield, or the appendage-less posterior trunk.

Fuxianhuiidae Hou and Bergström, 1997 [21].

### Diagnosis

Fuxianhuiids with subtrapezoidal head shield typically wider (trans.) than long (sag.), and covering (completely or partially) three small anteriormost trunk segments with reduced tergites in life position. Trunk subdivided into anterior appendage-bearing part with well-developed expanded tergopleurae encompassing between 50 to 60% of trunk length (sag.), and posterior appendage-less part with narrowed segments. Endopods of biramous appendages may feature rounded termination and no endites, or acute termination with developed ventral endites. Modified from [51, 52].

### Remarks

Our updated diagnosis of Fuxianhuiidae incorporates new observations of *Xiaocaris luoi* nov. from the present study, as well as the recent re-descriptions of *Guangweicaris spinatus* from the Cambrian (Stage 4) Guanshan biota [52, 53]. In this context, the unique characters that unite Fuxianhuiidae include the three small anteriormost tergites underneath the head shield, and a trunk subdivided in terms of its tergite morphology and presence of biramous appendages. Other relevant adjustments to the diagnosis of Fuxianhuiidae include: 1) the recognition that the head shield in all members of this group is wider than long, particularly when compared to that of Chengjiangocarididae [21] Hou and Bergström, 1997, in which the length/width ratio is approximately 1:1; 2) the dimensions of the anterior tergites with expanded tergopleurae, associated with appendage-bearing segments, comprise between 50 to 60% of the trunk length (sag.), whereas the appendage-bearing region may comprise up to 80% of the trunk length (sag.) in Chengjiangocarididae [5, 21, 51]; 3) the fact that the endopod morphology is variable between *Fuxianhuia* species (i.e. rounded tips and no endites) when compared with *Guangweicaris* (acute termination with ventral endites). It should be noted that taxa falling outside these clades, namely *Shankouia zhenghei* [47], and *Liangwangshania biloba* [46], also have a head shield that is wider than long, but these two taxa lack other features that define Fuxianhuiidae, namely the presence of three small segments with reduced tergites and the strong differentiation of the trunk. Chen and colleagues recently suggested the status of *Shankouia* as a junior synonym, and potential sexual dimorph, of *Liangwangshania* [54]. Regardless of whether these taxa represent one or two different species, their exoskeletal organization is distinctive enough to avoid confusion with members of either Fuxianhuiidae or Chengjiangocarididae. For instance, *Liangwangshania/Shankouia* feature a fused paddle-shaped tailspine, and their tergites with expanded tergopleurae extend to approximately 90% of the trunk length (sag.), compared with the 50% (e.g. *Guangweicaris*) to 60% (*Fuxianhuia*) observed in Fuxianhuiidae.

### *Xiaocaris* gen. nov.

#### Etymology

Derived from the Chinese Xiǎo (小) meaning small, as this represents the smallest fuxianhuiid species to date in terms of known body size, and the Latin *caris*, meaning shrimp, a common suffix used to denote euarthropod affinities.

#### Type species

*Xiaocaris luoi* nov.

#### Diagnosis

Small fuxianhuiid with boomerang-shaped head shield with deeply procurved posterior margin. Head shield associated with prominent eye-bearing anterior sclerite. Trunk divided into an appendage-bearing anterior region with nine tergites, and an appendage-less posterior part with six narrowed segments. First three segments small relative to rest of trunk, with reduced tergites partially covered by head shield. Last appendage-less segment carrying a pair of terminal tail flukes. Antennae elongate, consisting of at least 18 articles. Endopod consisting of at least 15 proximal articles each bearing a well-developed spine-like endite, and one acute terminal article. Exopod with short but densely packed marginal setae.

*Xiaocaris luoi* nov.

1999 *Jianshania furcatus* gen. et sp. nov. Luo et Hu, − [41] Plate 8, Fig. 1a, b.

2002 *Jianshania furcatus* (mistyped as *Jianshania forficula*) – [42] Plate 6, Fig. 3a, b.

#### Etymology

Named after Prof. Huilin Luo, in recognition of his work on the Chengjiang biota and the discovery of the holotype of this species (He-f-6-5-63/64), and generously facilitating access to the material for its formal restudy.

#### Type material

**Fig. 2:**
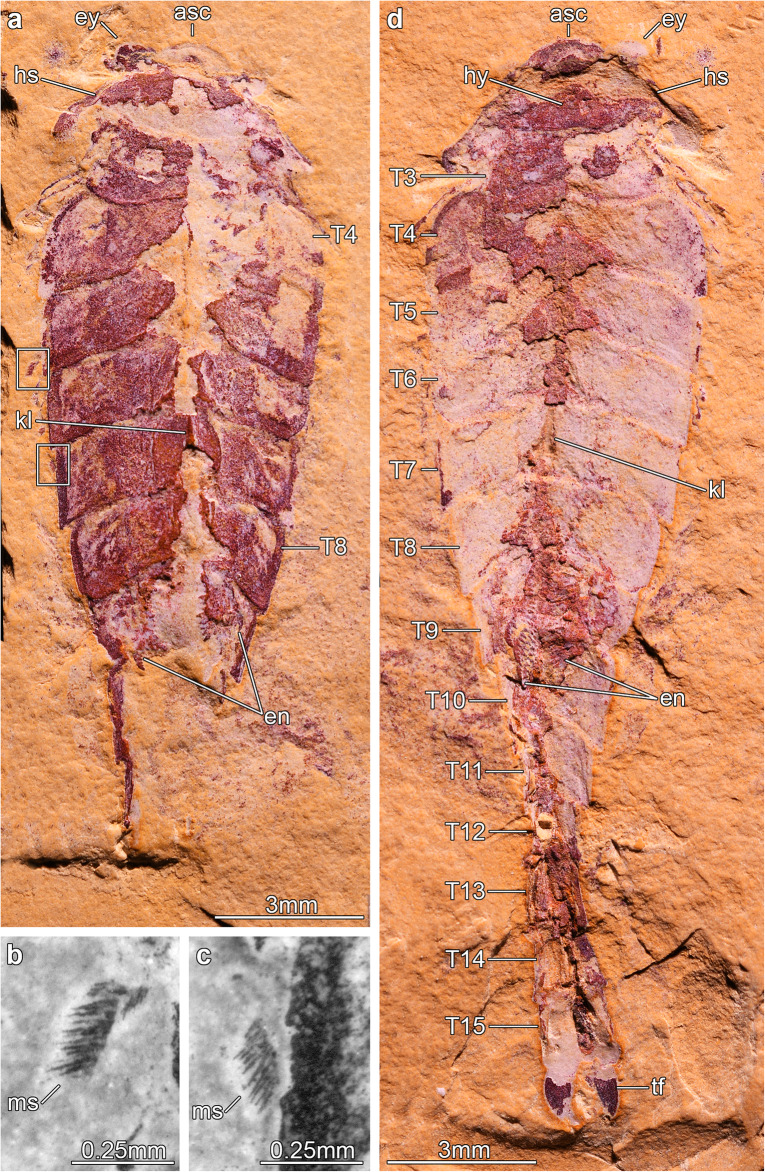
The fuxianhuiid *Xiaocaris luoi* nov. from the Ercaicun section of Haikou, Cambrian (Stage 3) Chengjiang biota. **a** Holotype (He-f-6-5-63), part of complete specimen preserved in dorsal–ventral aspect and photographed under cross-polarized light. **b** Magnification of an exopod underneath the fifth trunk tergite of He-f-6-5-63 showing marginal setae, photographed under fluorescent light. **c** Magnification of an exopod underneath the seventh trunk tergite of He-f-6-5-63 showing marginal setae, photographed with fluorescent light. **d** Holotype (He-f-6-5-64), counterpart photographed under cross-polarized light

**Fig. 3:**
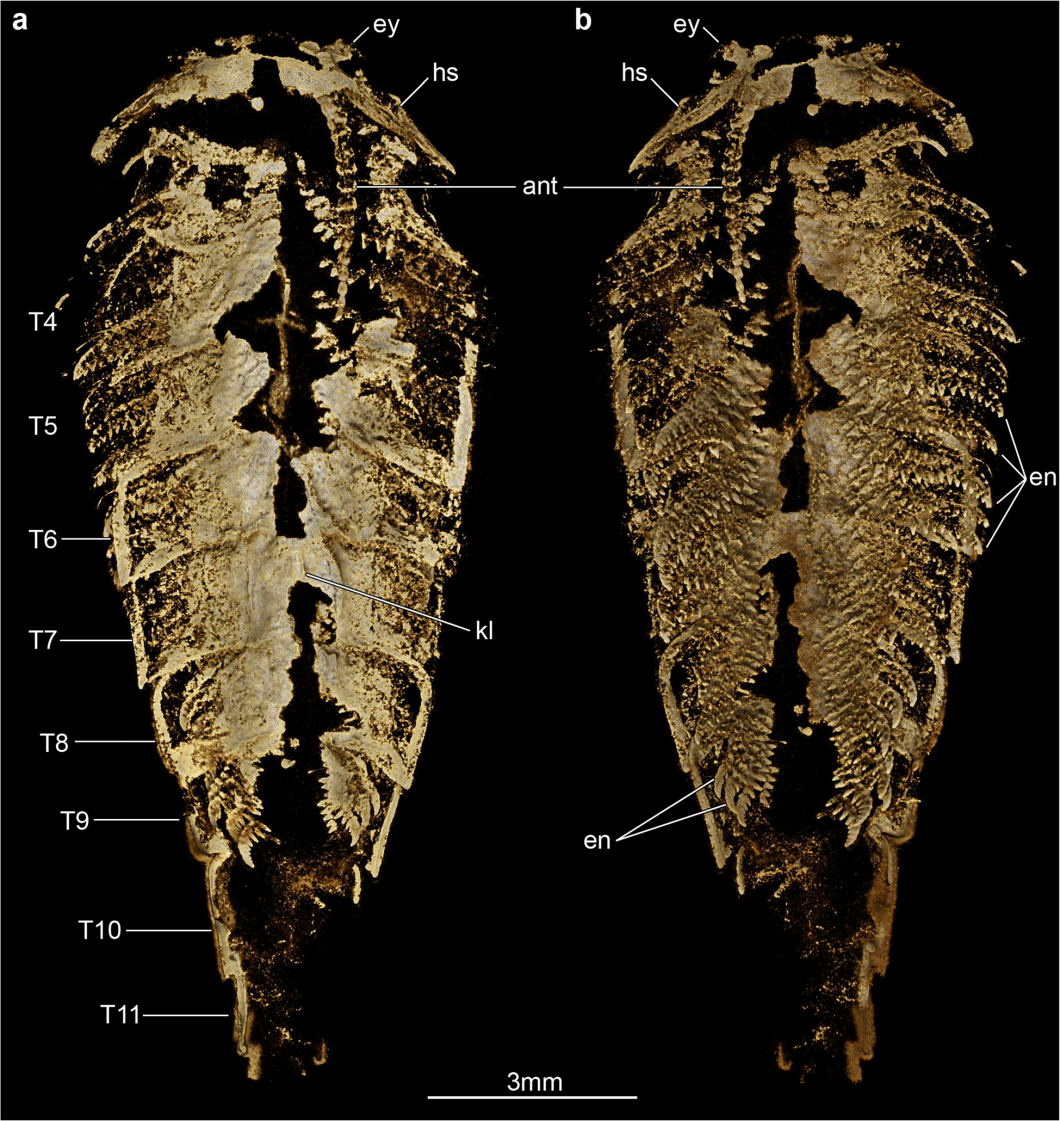
Overall three-dimensionally preserved exoskeletal morphology of *Xiaocaris luoi* nov. part (He-f-6-5-63, see also Fig. 2a). **a** Tomographic model of whole specimen in dorsal view. **b** Tomographic model of whole specimen in ventral view

He-f-6-5-63 (holotype, part) and He-f-6-5-64 (holotype, counterpart) of a complete individual (Figs. 2, 3). The specimen was identified as *Jianshania furcatus* in the original description of that species, along with the holotype (Fig 1). Chen and colleagues included part and counterpart of the same specimen in their Plate 6, Fig. 3a, b and noted that He-f-6-5-64 was the holotype of *Jianshania furcatus* [42]. However, this was incorrect, because the holotype of *J. furcatus* had already been designated as Hz-f-6-307 in the original description [41].

#### Locality and horizon

Ercaicun section in Haikou, Kunming, China. Yu’anshan Member, Chiungchussu Formation (Cambrian Stage 3), *Eoredlichia-Wutingaspis* trilobite biozone [12].

#### Diagnosis

As for genus.

#### Description

**Fig. 4:**
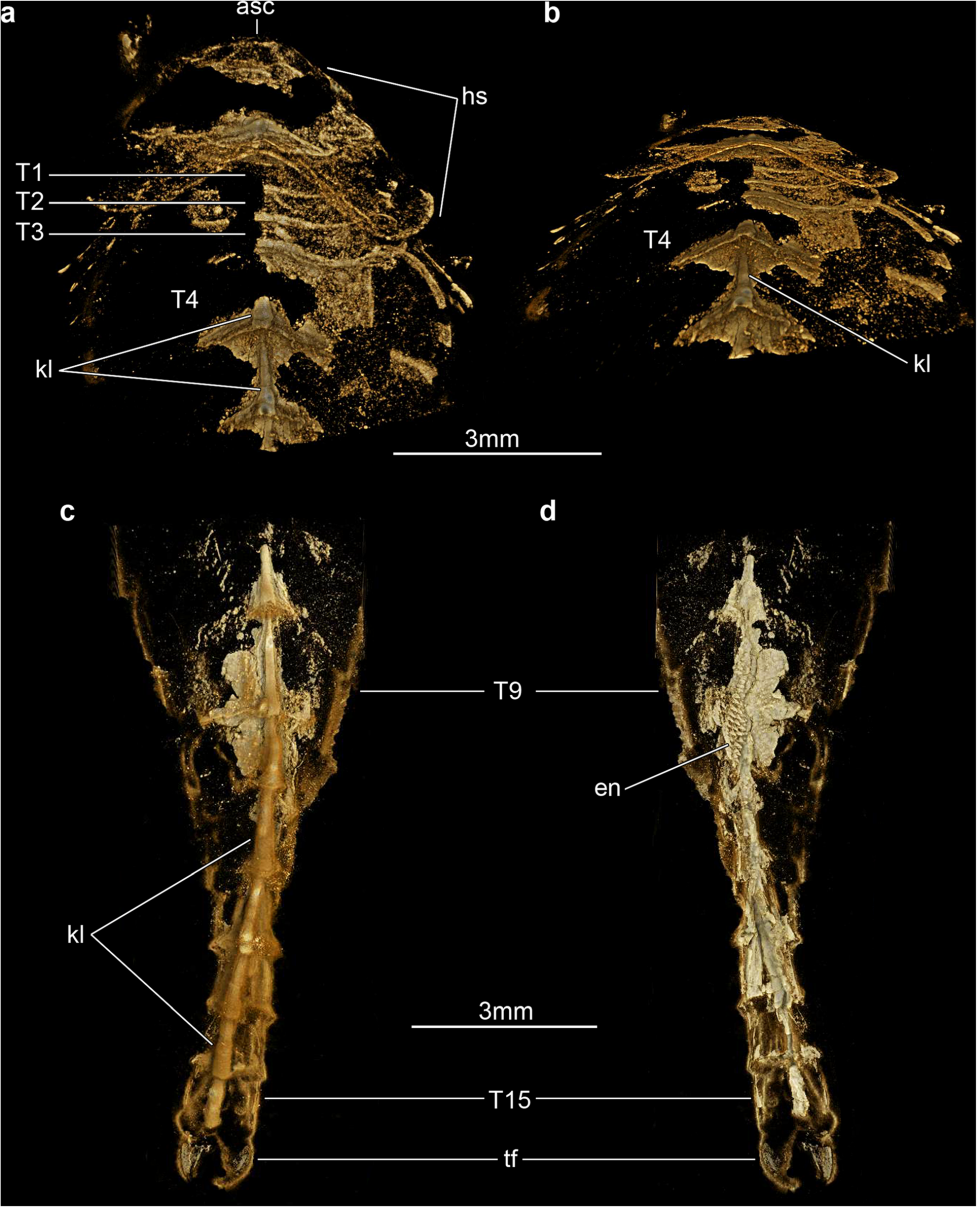
Three-dimensionally preserved exoskeletal morphology of *Xiaocaris luoi* nov. counterpart (He-f-6-5-64, see also Fig. 2d). **a** Tomographic model of anterior region in ventral view. **b** Same as panel (**a**) but with 60-degree inclination away from the observer. **c** Tomographic model of posterior trunk region in dorsal view. **d** Same as panel (**c**) but in ventral view

The only known specimen, He-f-6-5-63/64, designated as holotype herein, represents a completely articulated individual preserved in dorsal–ventral aspect, with a total length (sag.) of 20.5 mm, and a maximum width (trans.) of 5 mm, measured at the level of the fourth trunk tergite. The dorsal exoskeleton consists of a broad, semicircular anterior sclerite associated with stalked lateral eyes, and articulated to the anterior edge of a boomerang-shaped head shield (Figs. 2, 3). The head shield has an approximately 1:2 maximum length/width ratio, and features rounded lateral angles, and a deeply procurved posterior edge that partially covers at least three anteriormost small trunk tergites underneath (Fig. 4a, b). The tergites of the small anterior segments have rounded pleurae, and also a gently procurved posterior margin. The trunk presents three anteriormost small segments and additional 12 broadly overlapping tergites that progressively narrow in width (trans.) towards the posterior end of the body (Figs. 2, 3, 4). The fourth to ninth trunk tergites comprise approximately half of the total trunk length (sag.); they are characterized by a subtrapezoidal outline, have expanded tergopleurae with straight lateral margins and acute lateroposterior tips, and an anteriorly reflexed posterior margin (Figs. 2, 3). With the exception of the anteriormost small segments, the remaining trunk tergites feature a well-developed dorsal keel that conveys an elevated subtriangular transverse profile to the body (Figs. 2a; 4a-c). The dorsal keels of successive tergites telescope with each other (Fig. 4a, b). The tenth to fifteenth tergites, corresponding to the appendage-less posterior trunk have a subrectangular outline with a longer sagittal axis, are considerably narrower due to the absence of expanded tergopleurae, and terminate in a pair of tail flukes (Figs. 2d; 4c, d). The presence of a discrete telson cannot be resolved due to preservation.

**Fig. 5:**
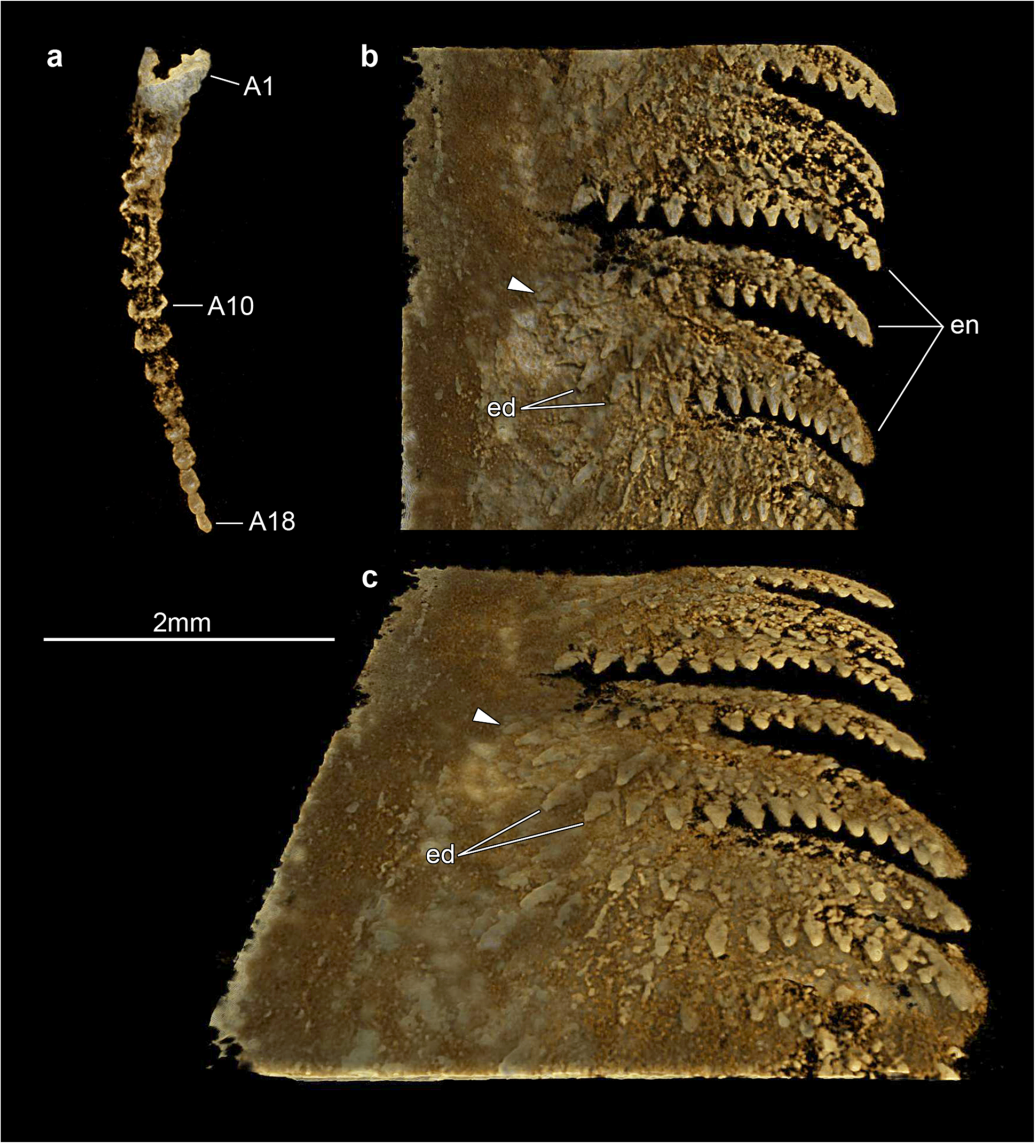
Appendage organization of *Xiaocaris luoi* nov. part (He-f-6-5-63). **a** Tomographic model of antenna in ventral view. **b** Tomographic model of trunk appendage series underneath fifth and sixth trunk tergites in ventral view. Arrowhead indicates position of most basal observable article. **c** Same as panel (**b**) but with 60-degree inclination away from the observer

Micro-CT reveals exceptional details of the ventral anatomy concealed within the rock matrix (Figs. 3, 4, 5). He-f-6-5-63 preserves the remains of elongate antennae composed of at least 18 articles, and attached close to the anterior edge of the head shield (Figs. 3a, b; 5a). It is possible to observe a fragmentary hypostome behind the antennae, as indicated by the presence of a bilobed posterior margin similar to that observed in other fuxianhuiids (Fig. 2d) [51]. The median position of the hypostome within the head shield suggests a slight degree of posterior displacement during burial. He-f-6-5-63 shows no other traces of preserved head appendages. Each of the three anteirormost small tergites corresponds to one pair of biramous appendage, whereas the following six tergites are associated with four pairs of biramous appendages that are densely packed, and slightly decrease in size towards the posterior end (Figs. 3b; 4d; 5b). There is no evidence for appendages associated with tergites 10–15. The endopod is composed of at least 15 articles that taper distally and bear a single well-developed triangular endite (Figs. 3b; 5b, c). The terminal claw is small and has an acute termination. Other than the presence of more robust endites towards the base of the appendage (arrowheads in Fig. 5b, c), there is no clear evidence for the presence of a differentiated protopodite in the trunk appendages. Densely arranged bundles of marginal spines preserved on the left side of He-f-6-5-63 demonstrate the presence of trunk exopods (Fig. 2b, c), but the structure of the flap is not preserved in this specimen.

### Remarks

Luo and colleagues originally assigned the specimen He-f-6-5-63/64 (*Xiaocaris luoi* nov. holotype; Fig. 2) to *Jianshania furcatus* (holotype: Hz-f-6-307, Fig. 1) [41]. Specimen He-f-6-5-63/64 was subsequently refigured by other workers under the incorrect name *Jianshania forficula* (nomen nudum) [42], but specimen Hz-f-6-307 was not illustrated again until now. Although *Jianshania* has been mentioned in various publications addressing the taxonomic composition of the Chengjiang biota, or comparisons between palaeobiogeographic patterns of soft-bodied euarthropods during the Cambrian [3, 12, 16, 22, 42], none of these studies addressed the morphology of the type material. Restudy of these specimens, together with the new insights on the exoskeletal organization of He-f-6-5-63/64 possible through micro-CT techniques, demonstrates that the material assigned to *Jianshania furcatus* belongs to two fundamentally different euarthropod taxa. Other than a superficial resemblance in terms of overall body shape and proportions, micro-CT indicates that He-f-6-5-63/64 can be assigned to Fuxianhuiida based on several diagnostic exoskeletal characters, most notably the presence of small trunk tergites under the head shield articulated to an eye-bearing anterior sclerite (Figs. 2, 3). These features are entirely lacking in the holotype of *Jianshania furcatus* (Hz-f-6-307, Fig. 1), which is characterized by a semicircular head shield with possible anterolateral notches accommodating large bulbous eyes, a straight posterior margin of the head shield, a trunk with 11 segments whose tergites taper and curve posteriorly, an undivided tailspine approximately one third the total body length (sag.) with one distal articulation, and a bifid termination. Hz-f-6-307 further differs from He-f-6-5-63/64 in the absence of an anterior sclerite, a lower trunk segment count, different tergite morphology without a dorsal keel, and presence of a tailspine rather than a narrowed trunk. These discrepancies support the assignment of He-f-6-5-63/64 as the holotype of *Xiaocaris luoi* nov, whereas Hz-f-6-307 remains as the holotype of *Jianshania furcatus*. We defer a more comprehensive reappraisal of the exoskeletal and ventral morphology of Hz-f-6-307 pending the availability of appropriately detailed micro-CT data currently under preparation.

## Discussion

### *Xiaocaris****luoi*** nov. as a member of Fuxianhuiidae

**Fig. 6:**
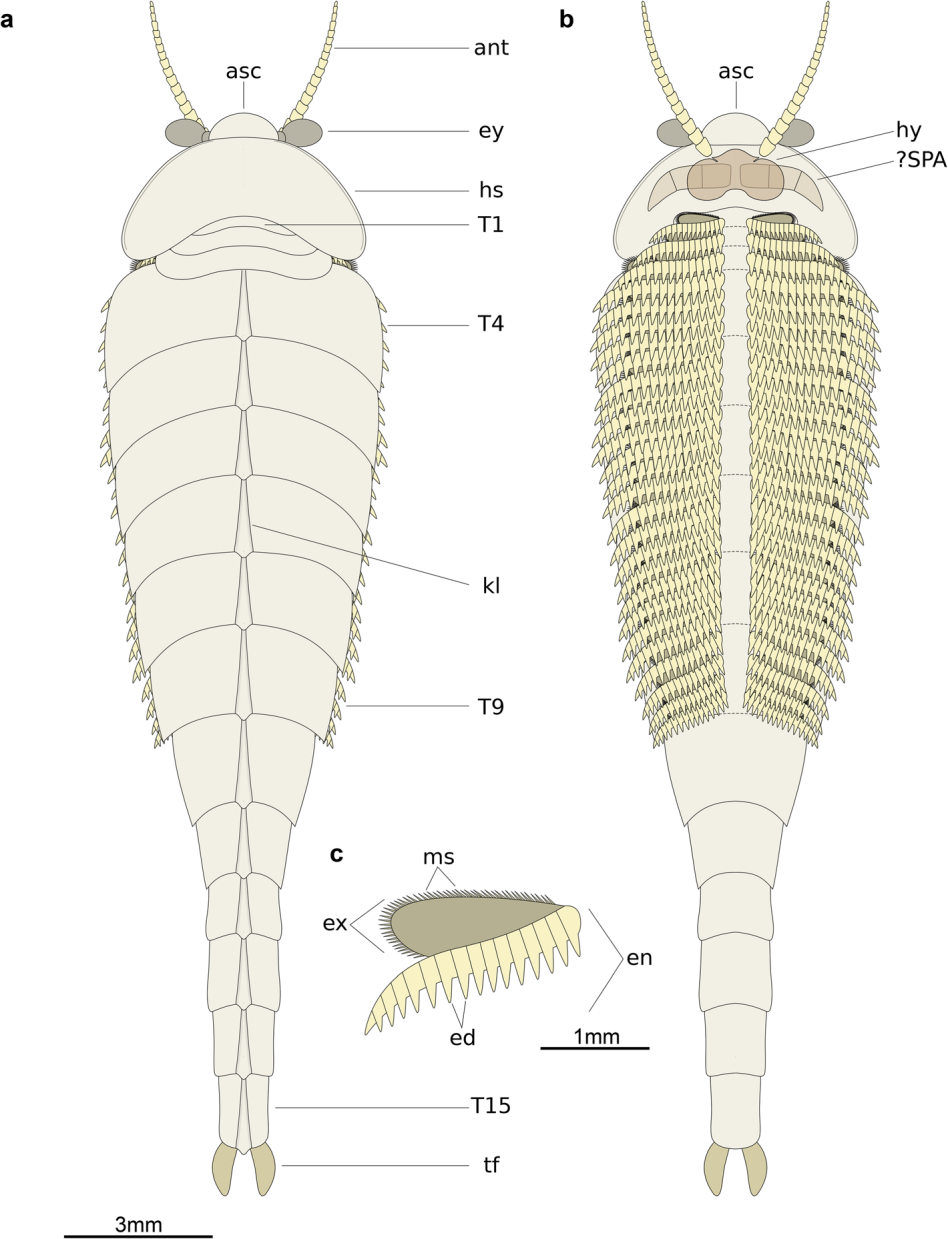
Morphological reconstruction of *Xiaocaris luoi* nov. **a** Dorsal view. **b** Ventral view; note that the anterior morphology of the hypostome, presence of specialized post-antennal appendages (SPA) and exopod structure are extrapolated from those of other better-known fuxianhuiids (see [51]). Dashed lines indicate position of posterior margin of tergites relative to ventral biramous appendages. **c** Biramous trunk appendage

**Fig. 7:**
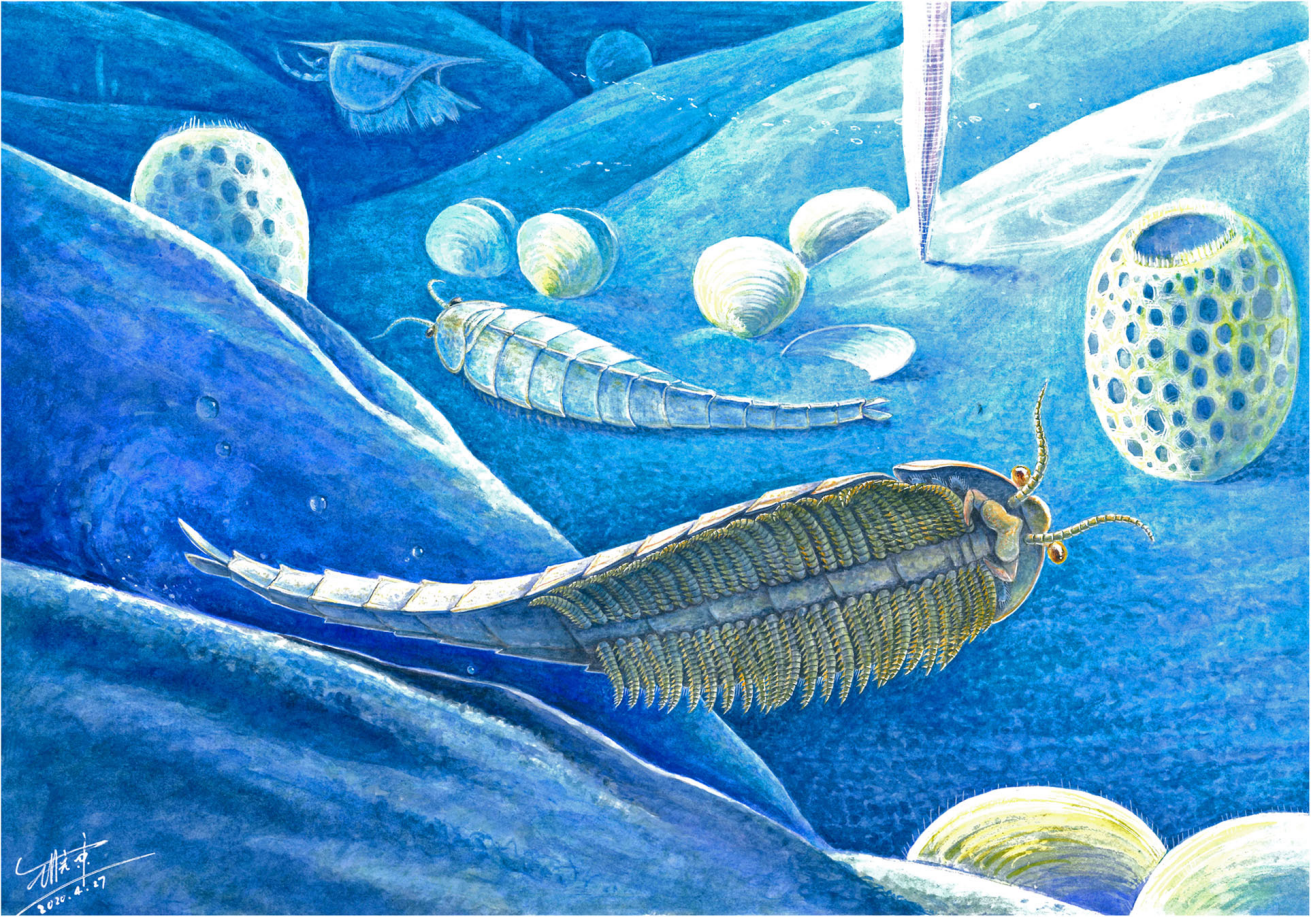
Morphological reconstruction of the fuxianhuiid *Xiaocaris luoi* nov. from the early Cambrian Chengjiang biota of South China. Artist: Mr. Xiaodong Wang

**Fig. 8:**
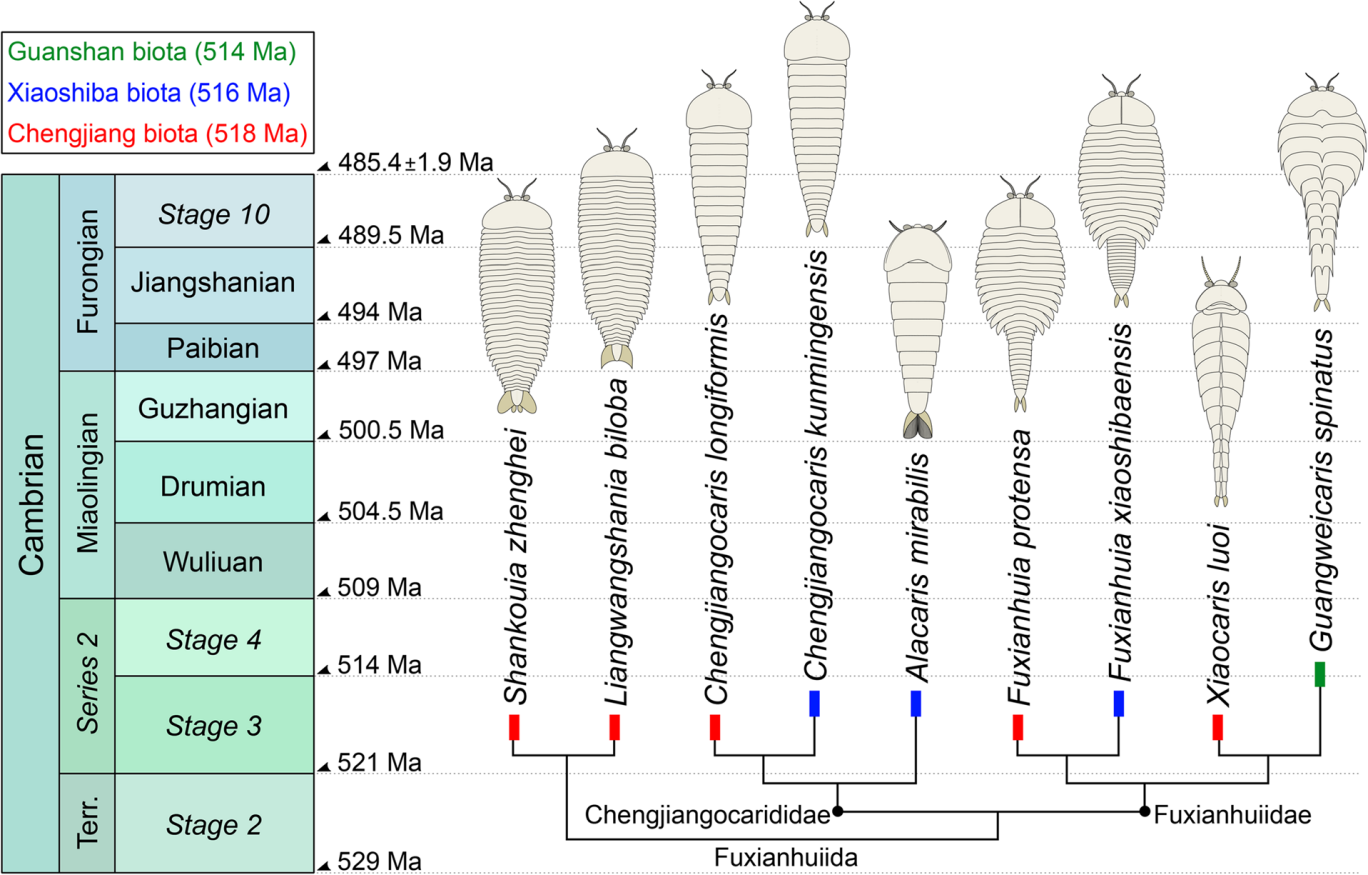
Stratigraphic distribution, diversity, and suggested phylogenetic relationships of fuxianhuiid euarthropods from the early Cambrian of South China. Overall tree topology follows results of [51]. Position of *Xiaocaris luoi* nov. based on characters shared with *Guangweicaris spinatus* (see Discussion). Modified from [55]

The exoskeletal and appendicular organization of *Xiaocaris luoi* nov. unequivocally identifies it as a representative of Fuxianhuiida [45] as presently defined (see also [5, 22, 51, 53, 54]), particularly given the diagnostic anteriormost reduced tergites under the head shield connected with an eye-bearing anterior sclerite [56], and the derived presence of multiple appendage pairs per trunk tergite, which also have endopods with over a dozen articles. Furthermore, the presence of at least three small tergites and a trunk consisting of an appendage-bearing anterior part, and an appendage-less, narrower posterior part, also represent diagnostic features of Fuxianhuiidae ([21], Figs. 6, 7, 8). In this context, *Xiaocaris* nov. increases the species richness and morphological diversity for Fuxianhuiidae, and further contributes towards reconstructing the relationships within this group.

*Xiaocaris* nov. most closely resembles *Guangweicaris spinatus* from the Guanshan biota [49, 52, 53, 57]. Both species share 15 trunk tergites with a single series of well-developed median tergal processes expressed as the strong dorsal spines throughout the trunk of *G. spinatus,* and the keel observed in *Xiaocaris luoi* nov. (Fig. 4). The structure of the endopods in these two taxa has an acute distal termination and well-developed ventral endites (albeit more robust in *Xiaocaris luoi* nov.) (Figs. 3; 5b, c) [52, 53], both of which contrast with the blunt endopods found in *Fuxianhuia* species [5, 21, 58], or the almost conical endopods of chengjiangocaridids [51, 59]. In the context of Fuxianhuiidae, *Xiaocaris* nov. also resembles *Guangweicaris* in having head shields with an approximately 1:2 length/width ratio, the subtrapezoidal outline of the tergites on the appendage-bearing part of the trunk, tergopleurae with straight lateral margins and subrectangular tergites of the appendage-less trunk region, all of which differ from the much shorter (sag.) head shield (1:4 length/width) and tergopleurae of *Fuxianhuia* [5, 21, 58]. *Xiaocaris* nov. is distinguished from *Guangweicaris* based on several characters, including: a deeper posterior notch in the head shield (Fig. 4a, b); the allocation of trunk tergites (nine appendage-bearing and six appendage-less segments in *Xiaocaris* nov. versus eight appendage-bearing and seven appendage-less ones in *Guangweicaris*) (Fig. 2); lack of developed tergal spines on the trunk; presence of four appendage pairs per trunk tergite in *Xiaocaris* nov., rather than two in *Guangweicaris* [52]. Notwithstanding these differences, it appears likely that *Xiaocaris* nov. and *Guangweicaris* may form a discrete clade within Fuxianhuiidae, as sister taxa to *Fuxianhuia* species (Fig. 8). *Xiaocaris* nov. shares some similarities with members of Chengjiangocarididae [5, 21, 51, 60], particularly the subtrapezoidal outline of the trunk tergites with straight tergopleural margins, the presence of up to four appendage pairs per tergite, and a more gradual posterior tapering of the body when compared with *Fuxianhuia* or *Guangweicaris*. *Xiaocaris* nov. specifically resembles *Alacaris* from the Cambrian (Stage 3) Xiaoshiba biota in overall appearance and in that the procurved posterior margin of the head shield is more pronounced in these taxa compared to all other fuxianhuiids (Fig. 4a, b) [51, 60], but differs in having a lower tergite count. Furthermore, all chengjiangocaridids are distinguished by a head shield with an approximately 1:1 length/width ratio, five anteriormost small tergites, and their endopods have an almost conical outline without developed ventral endites.

### Ontogeny and autecology of the smallest fuxianhuiid

*Xiaocaris* nov. holds the distinction of being the smallest member of Fuxianhuiida described to date (Table 1). With a total length (sag.) of 20.5 mm (Fig. 2), *Xiaocaris* nov. is six times smaller than the largest fuxianhuiid specimens known (e.g. [51]), and only directly comparable to juvenile individuals of *Fuxianhuia protensa* (see [31]) and *Chengjiangocaris kunmingensis* (see [5]). Given the substantial size differential, we reaffirm that *Xiaocaris* nov. represents a distinct genus, rather than a juvenile of another known taxon. For instance, the presence of three anteriormost tergites underneath the head shield makes *Xiaocaris* nov. only directly comparable with other members of Fuxianhuiidae. In euarthropods with an anamorphic phase of ontogenetic development, metameric segments are produced from a posterior growth zone and released towards the anterior end of the body, so that the segments and (their corresponding tergites) on the anterior half of the trunk will be ontogenetically older than those in the posterior half (e.g. [36, 61–63]). In this context, *Xiaocaris* nov. can be confidently discarded as a member of Chengjiangocarididae or *Shankouia*/*Liangwangshania* since all these forms have five or six anteriormost small segments with reduced trunk tergites that would have been patterned during early development. The drastic differences in size and morphology observed between the small anterior segments with reduced tergites (each associated with one appendage pair), and the larger tergites that constitute most of the trunk (each associated with multiple appendage pairs), suggest an important developmental and temporal shift during body elongation and dorsoventral segmentation in fuxianhuiids. Thus, we argue that the number of anterior reduced tergites represents a phylogenetically and developmentally meaningful character to distinguish between fuxianhuiid taxa, even if these euarthropods share a fundamentally similar overall pattern of trunk tagmosis. Within Fuxianhuiidae, it is also possible to discard the possibility of *Xiaocaris* nov. as a juvenile of *Fuxianhuia* thanks to the recent data on the hemianamorphic development of the latter [31]. *Fuxianhuia* juveniles are morphologically distinct from *Xiaocaris* nov. in the absence of a recurved posterior head shield margin, no dorsal keel, and differences in the proportions of the head shield and trunk tergites that only become more pronounced throughout *Fuxianhuia* ontogeny [31]. Finally, despite the close similarity between *Xiaocaris* nov. and *Guangweicaris*, these two can still be regarded as separate taxa. Whereas *Xiaocaris* nov. bears four pairs of biramous appendages per trunk tergite, *Guangweicaris* only features two pairs per tergite [52, 53]. Likewise, the appendage-less posterior trunk of *Xiaocaris* nov. presents six narrow tergites, but that of *Guangweicaris* contains seven. Considering that the trunk of both taxa consists of 15 tergites, it is highly unlikely that the appendages would shift in their number and distribution within the trunk simply as a result of an increase in body size during ontogeny, and particularly without the formation of additional body segments from the posterior end. Although we are restricted in our ability to ascertain whether He-f-6-5-63/64 represents a fully mature adult individual or not, the morphological differences relative to other closely related taxa consolidate its status as a separate fuxianhuiid species.

**Table 1:** Comparison between fuxianhuiid species sorted by maximum length in ascending order

Taxon	Locality/Age	Min. length (mm)	Max. length (mm)	Reference
*Xiaocaris luoensis* nov.	Chengjiang Stage 3	N/A	20.5	This study
*Fuxianhuia xiaoshibaensis*	Xiaoshiba Stage 3	28	32	[5, 51]
*Chengjiangocaris kunmingensis*	Xiaoshiba Stage 3	17	63	[5, 59]
*Liangwangshania biloba*	Chengjiang Stage 3	45	66.6	[54]
*Shankouia zhenghei*	Chengjiang Stage 3	50	75	[47]
*Fuxianhuia protensa*	Chengjiang Stage 3	12	80	[21, 31]
*Guangweicaris spinatus*	Guanshan Stage 4	60	95	[52, 53]
*Chengjiangocaris longiformis*	Chengjiang Stage 3	N/A	ca. 100	[21, 47]
*Alacaris mirabilis*	Xiaoshiba Stage 3	60	120	[51, 60]

The preserved functional morphology of *Xiaocaris* nov. reveals important aspects of its autecology, regardless of its small size and uncertain degree of ontogenetic maturity. The antennae are elongate and most likely had a chemo-tactile sensorial function that was further complemented by the large stalked lateral eyes (e.g. [26]). Well-preserved fuxianhuiids typically possess a pair of specialized post-antennal appendages (SPAs) located posterior to the antennae in a para-oral position, and which appear to have been involved in food manipulation based on their location, degree of motion, and overall robust constitution [5, 51, 52]. Although is not possible to observe these appendages in *Xiaocaris* nov. due to incomplete preservation, He-f-6-5-63/64 reveals instead an important degree of post-oral appendage complexity absent in most other fuxianhuiids. The biramous appendages of *Xiaocaris* nov. are densely packed throughout the trunk and form a well-defined median food groove that is aligned with the sagittal body axis, and consequently the expected position of the mouth opening underneath the hypostome (Figs. 3b, 6b). Each of the (at least) 15 endopod articles carries a well-developed triangular ventral endite, the latter of which are more robust towards the proximal appendage bases (Fig. 4b, c). There is no clear evidence of a differentiated protopodite as expressed in the anterior appendages of *Alacaris mirabilis* [51], but the spinose enditic armature of *Xiaocaris* nov. strongly suggests that the appendages were probably used in coordinated metachronal waves to secure, process and transport food items towards the mouth opening. Numerous Cambrian euarthropods feature similar feeding adaptations consisting of well-developed spinose endites directed adaxially to form a median food groove [38, 64], which suggests that *Xiaocaris* nov. was most likely a benthic scavenger. More specifically, we hypothesize that the high degree of appendage spinosity in *Xiaocaris* nov. would have been better suited for shredding soft food items or organic matter rather than durophagy, as the latter strategy typically requires the presence of a structurally robust and enlarged protopodite with strengthened endites for crushing prey [64–66].

The feeding adaptations observed in *Xiaocaris* nov. set it apart from the post-oral appendage morphology of most fuxianhuiids, which mainly consists of short or elongate endopods with a generally smooth or conical outline, and for the most part lacking in spinose elements (with the exception of *Guangweicaris* [52, 53];). Fuxianhuiid feeding autecology remains largely obscure, as most representatives either have mostly undifferentiated appendages with the exception of the SPAs (e.g. *Fuxianhuia, Chengjiangocaris*; see [5, 21, 55, 58, 59]), or the appendages themselves are simply poorly known (e.g. *Shankouia/Liangwangshania* [47, 54];). For example, a single enigmatic euarthropod from the Wuliuan Kaili Formation has been regarded as a putative fuxianhuiid with preserved gut contents [67], but the affinity of this fossil remains highly dubious owing to its poor preservation. *Alacaris mirabilis* from the Xiaoshiba biota is the only fuxianhuiid with a differentiated and enlarged protopodite with prominent gnathobasic spines, but only restricted to three pairs of post-oral appendages the anterior end of the body, whereas the endopods through most of the trunk have an almost conical construction with rounded ventral edges [51, 60]. In addition to *Alacaris*, specimens of *Chengjiangocaris* from the Xiaoshiba biota demonstrate the presence of minute medially directed spine-like projections on the bases on the post-oral appendages that form a food groove [51], but otherwise the endopods lack well-developed endites suggesting limited food processing capabilities. Earlier studies have pointed that fuxianhuiid guts are commonly filled with sediment, which prompted interpretations that these euarthropods were mainly deposit feeders (e.g. [21, 58]). However, the recent recognition of a well-preserved digestive tract with paired midgut diverticulae in *Fuxianhuia protensa* argues instead for a more complex feeding ecology [25], in line with other Cambrian euarthropods with similar digestive system adaptations for promoting macrophagy, carnivory, and efficient digestion (e.g. [24]). Although He-f-6-5-63/64 lacks information on the organization of the gut, the functional morphology of the biramous appendages is congruent with a macrophagous scavenging habitus, which becomes all the more significant when considering its small size. The differences in the ventral appendage armature observed between *Xiaocaris* nov. and other larger Chengjiang fuxianhuiids (i.e. *Fuxianhuia protensa*, *Chengjiangocaris longiformis*, *Shankouia/Liangwangshania*) could suggest a degree of niche partitioning based on body size that avoided direct competition for resources between closely related species living in the same environment. A similar pattern, albeit within an ontogenetic context, has been recently recognized for nektobenthic euarthropods from Chengjiang, including the megacheiran *Leanchoilia illecebrosa* [68], and the trilobitomorph *Naraoia spinosa* [38], in which juveniles feature different feeding adaptations and autecologies compared to the corresponding adults. Ultimately, the exceptional appendicular data observed in *Xiaocaris* nov. contribute towards a more complete understanding of fuxianhuiid functional morphology and possible ecological adaptations in the Chengjiang biota.

### Fuxianhuiid diversity in early Cambrian deposits from South China

*Xiaocaris* nov. brings the number of monospecific fuxianhuiid genera for the Chengjiang biota to five (or four depending on the status of *Shankouia/Liangwangshania* [54];). Although both *Fuxianhuia* and *Chengjiangocaris* have species represented in the stratigraphically younger Xiaoshiba biota, none of the fuxianhuiid-bearing localities in the Yangtze platform have more than one species of any given taxon (Fig. 8; Table 1). Thus, the Chengjiang biota contains the highest species diversity of fuxianhuiid euarthropods to date, followed by the Xiaoshiba biota with three [5, 51], and finally the Stage 4 Guanshan biota [52, 53]. Some broad patterns emerge from this temporal distribution. Stratigraphically younger fuxianhuiid species appear to become both less diverse and abundant in their respective localities. This is well exemplified by *Fuxianhuia*, which is known from hundreds of specimens in the Chengjiang (e.g. [3, 21, 58]), but less so in the Xiaoshiba [5, 6, 51], and is completely absent from Guanshan. Although *Chengjiangocaris* is better represented in the Xiaoshiba [5, 59] compared to Chengjiang [3, 21], this would appear to result from a lack of worker effort as *Chengjiangocaris longiformis* has not been thoroughly revised in over 20 years [21]. *Shankouia/Liangwangshania* are endemic to Chengjiang, but lack close relatives in any of the younger deposits based on the results of recent phylogenetic analyses (e.g. [51]). *Xiaocaris* nov. is unique within the group as it represents the rarest fuxianhuiid described to date, known from a single specimen, but also its closest relative is found in a much younger stratigraphic unit (i.e. *Guangweicaris* in Guanshan) without any known intermediates in the Xiaoshiba biota (Fig. 8). The discovery of *Xiaocaris* nov. points towards a more complex evolutionary history of Fuxianhuiida than previously considered, and suggests the existence of cryptic euarthropod species in early Cambrian deposits from the Yangtze platform with great promise for future discoveries through advanced imaging methods.

## Conclusions

We employed micro-CT techniques to restudy the fossil material originally ascribed to the enigmatic Chengjiang euarthropod *Jianshania furcatus*, and revealed previously unknown morphological details of the ventral appendage organization. The holotype of *J. furcatus* features a semicircular head shield with eye notches, stalked lateral eyes, a body with 11 segments and an elongate tailspine with a distal bifurcation. By contrast, we find that the second specimen initially assigned to *J. furcatus* represents a separate new taxon altogether, *Xiaocaris luoi* nov., based on the presence of 15 trunk tergites, including three small anterior segments with reduced tergites that are partially overlain by the head shield in life position. The morphology of the ventral appendages, including the presence of elongate antennae, endopods with 15 articles and robust endites, and multiple biramous appendage pairs under most trunk tergites strongly support fuxianhuiid affinities for *X. luoi* nov. The presence of 15 trunk tergites with a single series of well-developed median tergal processes keel and endopods with robust ventral endites in *X. luoi* nov. supports its sister-group relationship to *Guangweicaris spinatus* from the upper Guanshan biota. In turn, this phylogenetic relationship suggests a cryptic evolutionary radiation within Fuxianhuiidae. The well-developed endites of *X. luoi* nov. indicate a benthic scavenger mode of life, that most likely fed on soft food items or organic matter. The recognition of *Xiaocaris luoi* nov. demonstrates the potential of micro-CT to study the exceptionally preserved fossils of Chengjiang and promise for new discoveries. A thorough redescription of the morphology of the *Jianshania furcatus* holotype is currently in preparation.

## Methods

The studied material consists of two specimens from the Ercaicun section of Haikou, Kunming, Yunnan Province [12, 41]. Stratigraphically, they belong to the Yu’anshan Member, Chiungchussu Formation (Cambrian Stage 3). These include the *Jianshania furcatus* holotype, specimen Hz-f-6-307 (Fig. 1), and a second specimen He-f-6-5-63/64 (Figs. 2, 3, 4) that is herein re-described as a new euarthropod *Xiaocaris luoi* gen. et sp. nov.

The specimens were photographed under cross-polarized light and fluorescent illumination to document details of the preserved dorsal morphology. Digital photographs were captured with a Nikon D850 DSLR fitted with a 60 mm Nikkor macro, and fluorescence microscopic images were captured with a Leica DFC7000T CCD linked to a Leica Mz10 F fluorescent microscope. We employed X-ray micro-computed tomography (micro-CT) to study exoskeletal and ventral structures concealed within the rock matrix (e.g. [35–40]). X-ray scanning of the part (Figs. 3 and 5) was performed on a Zeiss X-radia 520 Versa (voltage 60 kV, current: 84 μA, voxel size: 6.59 μm) in the Institute of Geology and Geophysics, Chinese Academy of Sciences. ROI scans of the head and posterior trunk region (Fig. 4) were performed on a Zeiss X-radia 520 Versa (voltage 50 kV, current: 80 μA, voxel sizes: 6.17 μm, 4.49 μm) at the Yunnan Key Laboratory for Palaeobiology, Yunnan University. Each scan generated a set of radiographs saved as TIFF stacks which were further processed with the Drishti software (version 2.4) [69]. The 3D models rendered in Drishti were screen-captured as images in the figures, and were recorded as videos included in the supplementary material.


**Additional file 1: Video S1.** Dorsal side of *Xiaocaris luoi* nov. as shown in Fig. 3a.



**Additional file 2: Video S2.** Ventral side of *Xiaocaris luoi* nov. as shown in Fig. 3b.



**Additional file 3: Video S3.** Head of *Xiaocaris luoi* nov. as shown in Fig. 4a, b.



**Additional file 4: Video S4.** Head of *Xiaocaris luoi* nov. as shown in Fig. 4c, d.


## Data Availability

The material is deposited in the Yunnan Institute of Geological Survey (He-f-6-5-63/64, Hz-f-6-307a, b). Videos supporting this article have been uploaded as supplementary material.

## Abbreviations

ant: Antenna
asc: Anterior sclerite
bt: Bifurcate termination
ed: Endite
en: Endopod
ey: Stalked eye
hs: Head shield
hy: Hypostome
kl: Dorsal keel
ms: Marginal setae
A*n*: The *n*th article
tf: Tail fluke
T*n*: The *n*th trunk tergite/segment
tp: Tailspine
